# A Simple Ionic-Gelation Method for Chitosan Nanoparticle Synthesis and Standardized Protocols for Biological Safety Assessment: Antibacterial Activity, Phytotoxicity, and Biocompatibility

**DOI:** 10.3390/ijms27083673

**Published:** 2026-04-20

**Authors:** Kanchit Rahaeng, Atcha Oraintara, Wuttipong Mahakham

**Affiliations:** 1 Department of Biology, Faculty of Science, Khon Kaen University, Khon Kaen 40002, Thailand; kanchit.ra@kkumail.com; 2 Department of Microbiology, Faculty of Science, Khon Kaen University, Khon Kaen 40002, Thailand; atcha@kku.ac.th

**Keywords:** chitosan nanoparticles, ionic gelation, standardized bioassays, antibacterial activity, seed germination assay, cell viability, human skin cells, biosafety assessment

## Abstract

Chitosan nanoparticles (Ch NPs) are versatile nanomaterials with expanding agricultural and biomedical applications, highlighting the need for reproducible, low-cost, and scalable synthesis methods to ensure their safe and widespread use in biological systems. This study presents a simple ionic-gelation protocol using a serological pipette–needle dropwise system that minimizes reagent waste and requires no sophisticated equipment. The synthesized Ch NPs were characterized by UV–Vis spectroscopy, ESEM, TEM, EDS, DLS, XRD, and FTIR, confirming nanoscale size, strong positive surface charge, and characteristic chitosan–TPP interactions. To establish a standardized biological safety assessment framework, three representative bioassays were implemented across microbial, plant, and mammalian systems. Antibacterial testing against *Xanthomonas oryzae* pv. *oryzae* (*Xoo*) using a resazurin-based microdilution assay revealed a minimum inhibitory concentration (MIC) of 128 µg/mL, whereas bulk chitosan showed no inhibition up to 512 µg/mL. Phytotoxicity and seed germination assays on rice (*Oryza* ‘KDML105’) demonstrated no inhibitory effects on germination, with over 90% germination by day 3 and significantly enhanced seedling growth parameters (*p* < 0.05) at 64–128 µg/mL, indicating non-phytotoxicity. MTT assays confirmed that Ch NPs were non-toxic to both human skin cell lines (HDF and HaCaT) across 2.5–160 µg/mL, showing enhanced cell viability in HDF cells at specific concentrations and stable viability in HaCaT cells, indicating overall biocompatibility. Importantly, all bioassays were conducted under aligned concentration ranges to enable cross-system comparison and reproducibility. This integrated workflow links nanoparticle synthesis with a standardized, multi-system evaluation strategy, supporting the safe application of Ch NPs in biological systems.

## 1. Introduction

Chitosan (CS) is a naturally derived polysaccharide obtained by the partial deacetylation of chitin, a major structural component in crustacean shells. Owing to its excellent physicochemical and biological properties, including biocompatibility, biodegradability, low toxicity, and intrinsic antimicrobial activity, chitosan has emerged as a versatile biomaterial with broad applications in biomedical, pharmaceutical, and agricultural fields [1,2]. In particular, chitosan nanoparticles (Ch NPs) exhibit enhanced physicochemical and biological functionalities compared with bulk chitosan (Bulk Ch), including increased surface area, improved colloidal stability, tunable particle size, and controlled release capabilities, which collectively improve their performance in biological systems [1,2,3]. These improvements are primarily attributed to their nanoscale dimensions and surface charge, which facilitate stronger interactions with biological interfaces and enhance functional efficiency. As a result, Ch NPs have emerged as a versatile nanoplatform for a wide range of applications, including antimicrobial systems [4], drug delivery [2,5], wound healing [6] and agricultural applications such as plant protection and growth regulation [7,8], where controlled release and stability are critical for effective performance [1,3].

Several preparation methods have been developed to produce Ch NPs with desired properties, such as ionic gelation, emulsification–solvent evaporation, spray drying, self-assembly, and desolvation [2,3,9]. Among these, the ionic gelation method, which relies on the electrostatic interaction between protonated amino groups of chitosan and negatively charged tripolyphosphate (TPP) ions, is the most widely employed due to its mild, solvent-free, and eco-friendly conditions [10,11,12,13]. However, traditional ionic-gelation setups often depend on peristaltic pumps or automated droplet systems to control the TPP addition rate, increasing equipment costs and limiting reproducibility between laboratories [11,14,15]. As a result, there remains a critical need for a low-cost and reproducible synthesis protocol that maintains nanoparticle uniformity while minimizing reagent waste and instrumental complexity.

Beyond their preparation, Ch NPs have been extensively studied for their broad-spectrum antimicrobial activity, particularly against both Gram-positive and Gram-negative bacteria [1,4,16]. The antibacterial mechanisms are generally attributed to electrostatic interactions between positively charged amino groups and negatively charged bacterial cell membranes, leading to membrane disruption, leakage of intracellular contents, and inhibition of vital enzymatic processes [1,4,17]. Recent studies have highlighted the efficacy of Ch NPs and chitosan–metal nanocomposites (e.g., chitosan–Fe nanocomposites) against *Xanthomonas oryzae* pv. *oryzae* (*Xoo*), the causative agent of rice bacterial leaf blight [18,19,20]. These findings underscore the potential of chitosan-based nanomaterials as sustainable biocontrol agents in plant disease management.

In addition to their antimicrobial activity, Ch NPs have demonstrated broad biocompatibility across different biological systems. In the biomedical field, Ch NPs have been widely explored for applications such as drug delivery, tissue engineering, dental materials, and wound-healing systems due to their biocompatibility, biodegradability, and hemostatic properties [6,21,22,23]. Similarly, in agricultural systems, Ch NPs have been reported to function as both bioactive elicitors and nanocarriers, contributing to improved seed germination, enhanced seedling growth, and modulation of plant physiological responses [7,8,24,25,26,27,28]. Their ability to act as carriers for bioactive compounds further supports controlled release and improved bioavailability in plant systems [8,24,25]. This cross-disciplinary applicability highlights the versatility of Ch NPs as a bridge between green nanotechnology and biologically compatible materials for both biomedical and agricultural applications.

Despite these advances, systematic and standardized frameworks for the biosafety evaluation of Ch NPs remain limited. Most studies have focused on individual aspects of nanoparticle performance, such as antimicrobial activity [29,30,31] or cytotoxicity [13,32], often assessed independently under varying experimental conditions. This fragmented approach hinders direct comparison across studies and limits the comprehensive understanding of nanoparticle safety and functionality across different biological systems. In particular, integrated evaluations that simultaneously consider microbial inhibition, plant responses, and mammalian cell compatibility under unified experimental conditions are rarely reported. Therefore, there is a critical need to establish reproducible and standardized evaluation protocols that enable cross-system comparison of Ch NP performance and safety.

In this study, we propose a simple, low-cost, and reproducible ionic gelation protocol for the synthesis of Ch NPs using an accessible dropwise addition system that eliminates the need for specialized instrumentation. More importantly, a standardized biological evaluation framework was developed to assess nanoparticle performance across multiple biological systems, including antibacterial activity against *Xoo*, phytotoxicity and seed germination in rice plants, and biocompatibility in human skin cell lines, including human dermal fibroblasts (HDF) and the human keratinocyte (HaCaT) cell line. By integrating synthesis and multi-system biosafety assessment within a unified experimental design, this study provides a practical and scalable platform for evaluating chitosan-based nanomaterials, supporting their safe and sustainable application in agricultural and biomedical contexts.

## 2. Results

### 2.1. Synthesis and Characterization of Ch NPs

**Figure 1:**
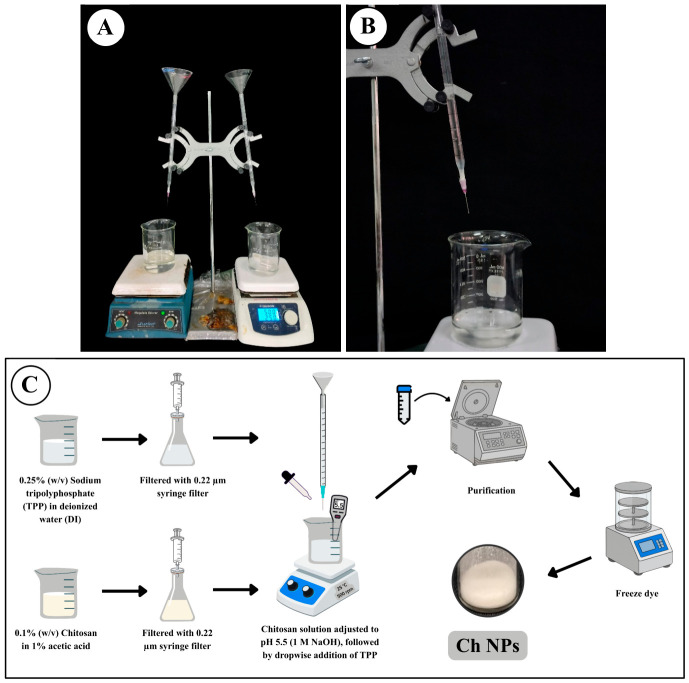
Schematic illustration of the simplified ionic gelation method for chitosan nanoparticle (Ch NP) synthesis: (**A**) Experimental setup of the manual dropwise addition system using a serological pipette–needle assembly; (**B**) Practical laboratory setup showing the dropwise addition of TPP solution into the chitosan solution under continuous stirring; (**C**) Schematic diagram illustrating the ionic gelation process and nanoparticle formation via electrostatic interaction between chitosan and TPP. Arrows indicate the sequential steps of the synthesis process.

Ch NPs were successfully synthesized using a simple and low-cost ionic gelation approach, as illustrated in Figure 1. The method employed a serological pipette–needle dropwise system, enabling controlled addition of TPP solution into the chitosan solution under continuous stirring at room temperature. This process generated a uniform milky suspension within minutes and required no peristaltic pump or advanced instrumentation, demonstrating a reproducible and accessible technique for nanoparticle formation. The practical laboratory setup of the dropwise ionic gelation system is shown in Figure 1A,B, while the overall synthesis workflow of Ch NPs is illustrated schematically in Figure 1C.

**Figure 2:**
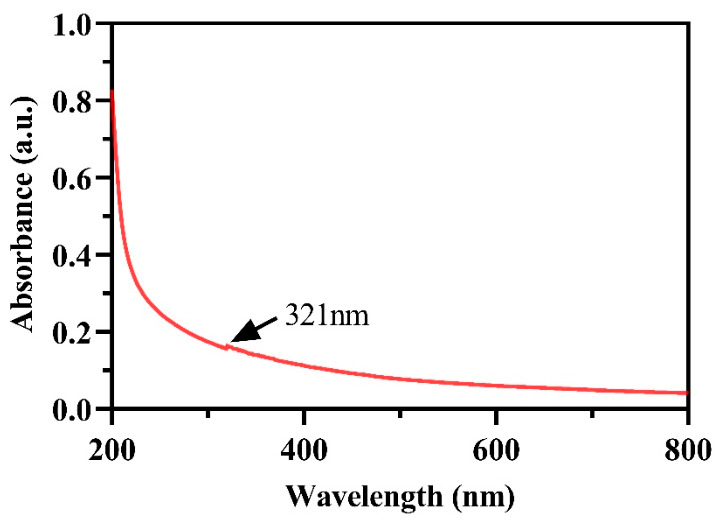
UV–Vis absorption spectrum of synthesized Ch NPs. A broad absorption band with a weak shoulder around 321 nm confirms the formation of chitosan–TPP complexes during ionic gelation.

**Figure 3:**
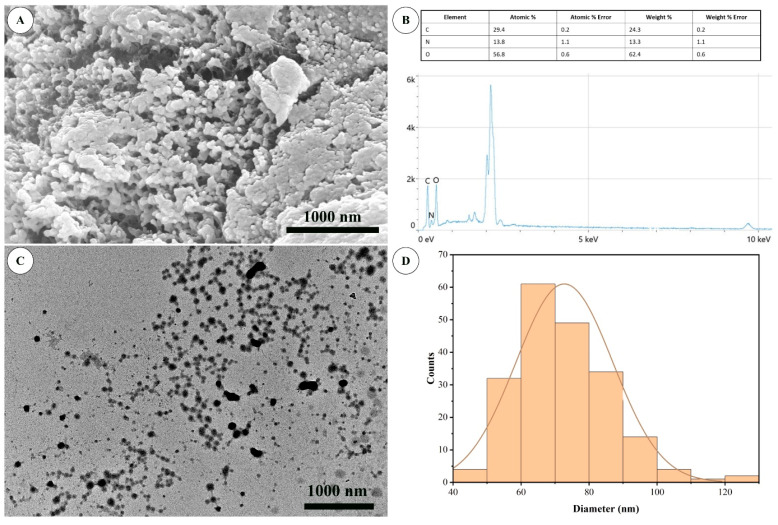
Morphological and structural characterization of Ch NPs: (**A**) Environmental SEM micrograph showing surface agglomeration; (**B**) EDS spectrum showing elemental composition; (**C**) TEM image showing spherical nanoparticles; (**D**) Particle size distribution histogram obtained from TEM, with the solid line representing the fitted Gaussian distribution.

The UV–Vis spectrum (Figure 2) exhibited a broad absorption band with a weak shoulder at approximately 321 nm, confirming the formation of chitosan–TPP complexes. Environmental scanning electron microscopy (ESEM) micrographs (Figure 3A) revealed aggregated nanoparticle clusters, while TEM images (Figure 3C,D) showed well-dispersed, nearly spherical particles with an average diameter of 75 ± 15 nm. Energy-dispersive X-ray spectroscopy (EDS) analysis (Figure 3B) identified carbon (29.4 at%), nitrogen (13.8 at%), and oxygen (56.8 at%) as the main elements, consistent with the composition of chitosan.

**Figure 4:**
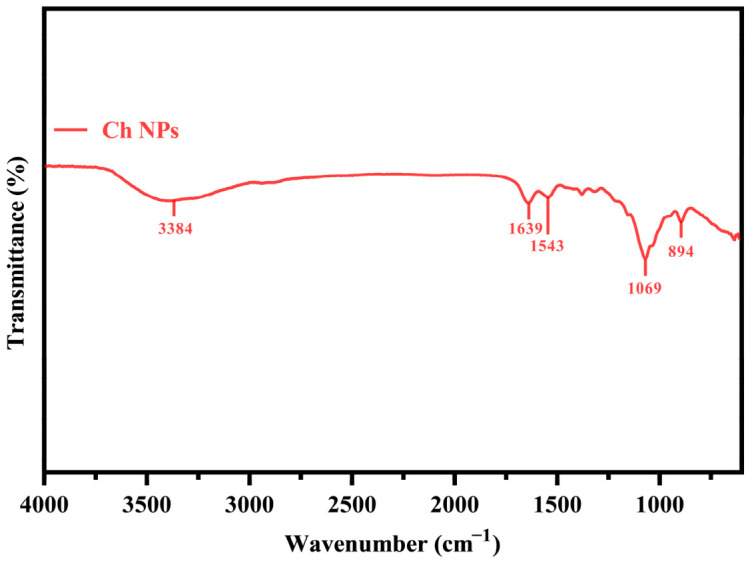
FTIR spectra of Bulk Ch and Ch NPs. Characteristic bands corresponding to chitosan functional groups and new phosphate-related peaks confirm ionic crosslinking between chitosan and TPP.

**Figure 5:**
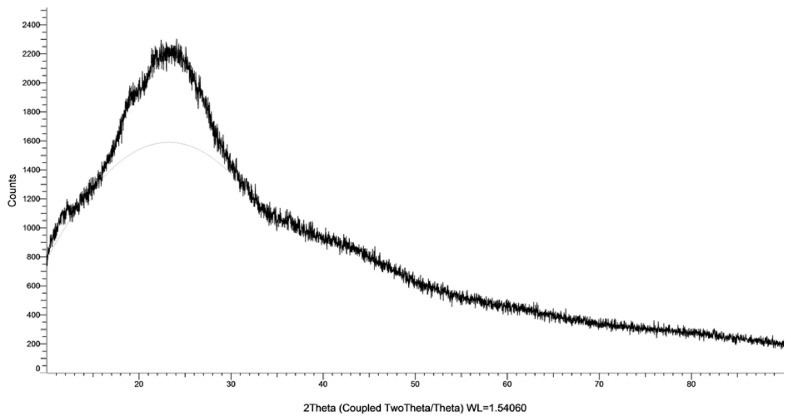
X-ray diffraction (XRD) pattern of Ch NPs. The broad diffraction halo centered around 2θ ≈ 20° indicates the predominantly amorphous structure of the nanoparticles after ionic gelation. The gray line represents the fitted/smoothed curve of the XRD pattern.

DLS measurements (Table 1) indicated a mean hydrodynamic diameter of 450.8 ± 8.75 nm and a polydispersity index (PDI) of 0.45 ± 0.02, suggesting moderate size uniformity. The zeta potential of +47.37 ± 1.01 mV confirmed the strong positive surface charge and colloidal stability of the synthesized nanoparticles. The FTIR spectrum (Figure 4) displayed characteristic bands of chitosan–TPP crosslinking at 3384 cm^−1^ (O–H/N–H stretching), 1639 cm^−1^ (amide I), 1543 cm^−1^ (amide II), 1069 cm^−1^ (C–O stretching), and 894 cm^−1^ (P–O asymmetric stretching). The XRD pattern (Figure 5) showed a broad diffraction halo centered near 2θ = 20°, indicating the amorphous structure of the Ch NPs.

### 2.2. Antibacterial Activity of Ch NPs Against Xoo

The antibacterial activity of Ch NPs was evaluated using a standardized resazurin-based microdilution assay to determine the minimum inhibitory concentration (MIC) against the bacterial leaf blight pathogen, *Xoo*. The assay relies on the colorimetric change in the redox indicator resazurin, which transitions from blue (oxidized form) to pink (reduced form) in response to bacterial metabolic activity. Thus, wells that remained blue indicated bacterial growth inhibition, while those turning pink reflected active bacterial metabolism.

**Figure 6:**
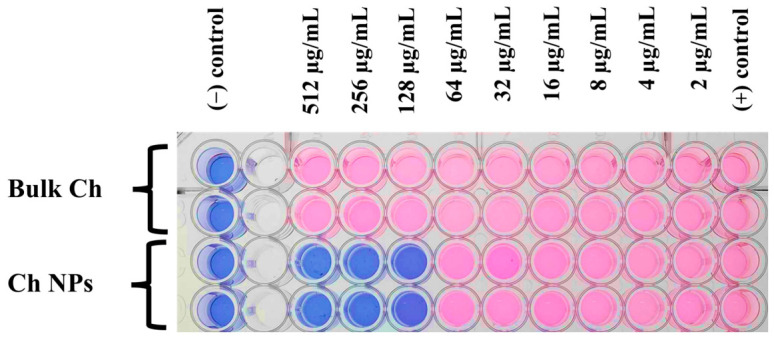
Resazurin-based microdilution assay for determining the minimum inhibitory concentration (MIC) of Ch NPs against *Xoo*. Blue wells indicate inhibition of bacterial metabolic activity, whereas pink wells represent active bacterial growth. The MIC was determined visually as the lowest concentration at which the wells remained blue, indicating inhibition of bacterial metabolic activity. The MIC of Ch NPs was determined to be 128 µg/mL.

As shown in Figure 6, color transitions were observed across the concentration gradient of Ch NPs (2 to 512 µg/mL). The MIC was determined at 128 µg/mL, representing the lowest concentration where wells remained blue with no visible bacterial growth. The negative control (sterile PSB medium with resazurin) remained blue, confirming the absence of contamination, while the positive control (untreated *Xoo*) turned uniformly pink, indicating full metabolic activity. In contrast, Bulk Ch exhibited no inhibition up to 512 µg/mL, confirming that nanoparticle formulation markedly enhanced antibacterial performance due to improved surface reactivity and charge-mediated interactions.

### 2.3. Phytotoxicity and Seed Germination Assay 

The effects of Bulk Ch and Ch NPs on rice seed germination and early seedling growth were evaluated using an agar-based assay. Seeds were pre-soaked in Bulk Ch or Ch NP suspensions at 0.5× MIC (64 µg/mL) and 1× MIC (128 µg/mL) for 24 h, followed by incubation on semi-solid agarose (0.4% *w*/*v*) medium under controlled growth conditions. These concentrations were selected based on the antibacterial MIC to ensure plant exposure did not exceed effective antimicrobial levels.

**Figure 7:**
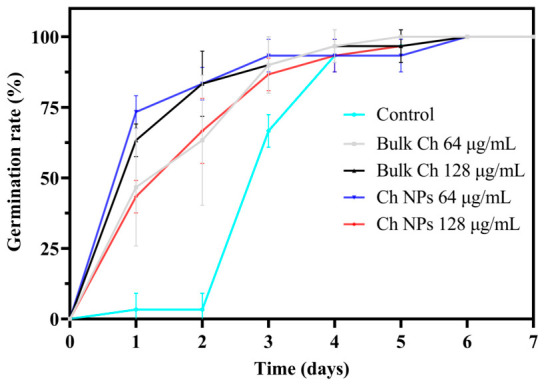
Germination rate of rice (*Oryza* ‘KDML105’) seeds treated with Bulk Ch and Ch NPs at 64 and 128 µg/mL. Seeds were incubated on semi-solid agarose (0.4% *w*/*v*) medium for 7 days in the dark at 28 °C. Ch NP–treated seeds exhibited faster and more uniform germination compared with the untreated control.

As shown in Figure 7, both Bulk Ch and Ch NP-treated seeds exhibited faster germination compared with the control, reaching over 90% germination by day 3, whereas the control required up to day 5 to achieve similar levels.

Quantitative germination parameters are summarized in Table 2. Germination percentage (GP) was 100% across all treatments, indicating no inhibitory effects on seed viability. Germination energy at day 3 (GE_3_) was significantly higher in all treated groups than in the control (*p* < 0.05), suggesting enhanced early germination activity.

Germination speed parameters, including mean germination time (MGT), time to 50% germination (T_50_), germination index (GI), germination velocity (GVe), and germination value (GV), were significantly improved in treated groups compared with the control (*p* < 0.05). Treated seeds exhibited reduced MGT and T_50_ values alongside increased GI, GVe, and GV, indicating faster and more synchronized germination. Comparable improvements were observed across all Bulk Ch and Ch NP treatments, with no significant differences among treatments.

**Figure 8:**
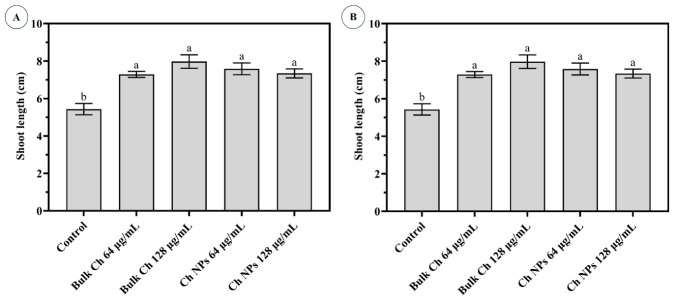
Growth of rice seedlings showing (**A**) shoot length and (**B**) root length after treatment with different concentrations of Bulk Ch and Ch NPs, compared with the control. Error bars represent the standard error (n = 3). Different letters indicate statistically significant differences according to Duncan’s test (*p* < 0.05).

Seedling vigor parameters were influenced by both treatment type and concentration (Table 2). Seedling length and seed vigor index (SVI) were significantly higher in BC 128 µg/mL and both Ch NP treatments than in the control (*p* < 0.05), whereas BC 64 µg/mL did not differ significantly from the control. The shoot-to-root ratio (SL/RL) showed a concentration-dependent pattern, with relatively higher values at 64 µg/mL than at 128 µg/mL. These trends are consistent with the increased shoot and root elongation observed in Figure 8.

**Figure 9:**
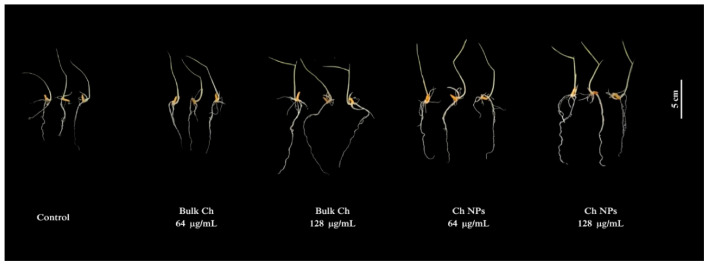
Representative images of rice seedlings (*Oryza* ‘KDML105’) after treatment with Bulk Ch and Ch NPs at 64 and 128 µg/mL for 3 days. All treatments showed normal seedling morphology without visible phytotoxic symptoms. Scale bar = 5 cm.

No visible phytotoxic symptoms, such as discoloration or growth inhibition, were observed in any treatment (Figure 9), indicating that both Bulk Ch and Ch NPs did not induce detectable phytotoxic effects under the tested conditions.

### 2.4. Biocompatibility Assessment in Human Skin Cells

**Figure 10:**
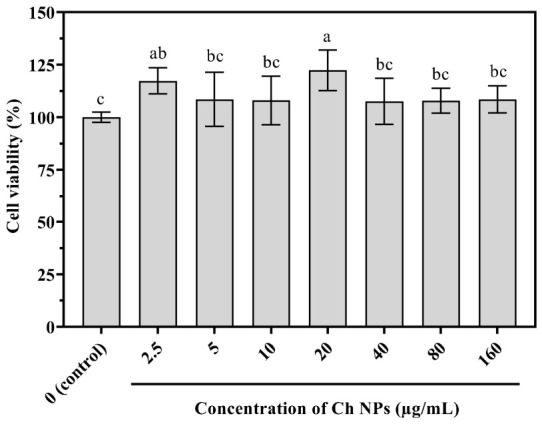
Cell viability of HDF cells treated with Ch NPs at concentrations of 2.5–160 µg/mL for 24 h. Viability was determined using the MTT assay and expressed as mean ± SE (n = 3). Different letters indicate statistically significant differences (*p* < 0.05).

The biocompatibility of Ch NPs was assessed using two human skin cell line models (i.e., HDF and HaCaT) through the MTT assay after 24 h of exposure to different concentrations (2.5–160 µg/mL) compared to the control (no Ch NPs). As shown in Figure 10, HDF cells exhibited a significant increase in viability at 20 µg/mL, reaching approximately 125% relative to the control (*p* < 0.05). All tested concentrations maintained viability values above 100%, indicating no cytotoxic effects on fibroblasts.

**Figure 11:**
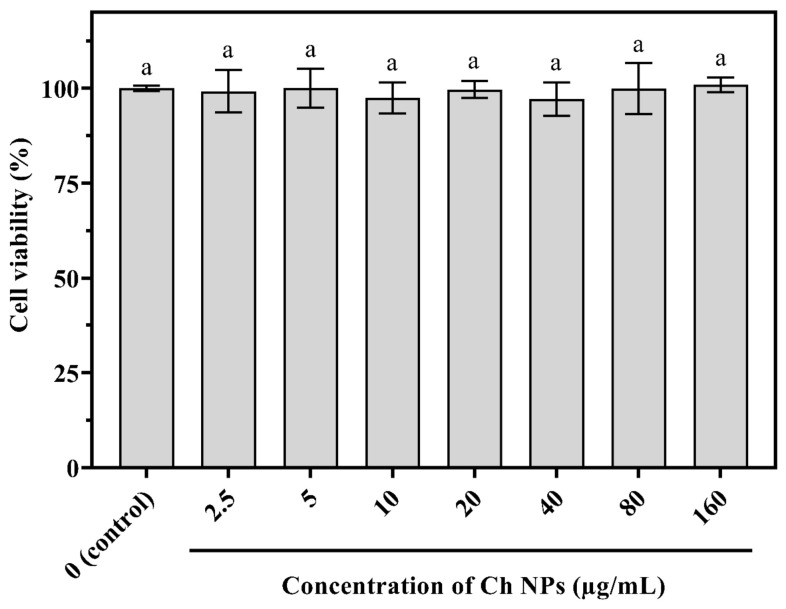
Cell viability of HaCaT cells treated with Ch NPs at concentrations of 2.5–160 µg/mL for 24 h. No significant differences were observed among treatments (*p* > 0.05), indicating non-cytotoxicity. The same letter (a) indicates no statistically significant difference among treatments (*p* > 0.05).

For HaCaT cells, no significant differences were detected among treatments (Figure 11). Cell viability remained close to 100% across all concentrations, confirming non-cytotoxicity and stable metabolic activity in keratinocytes.

## 3. Discussion

### 3.1. Synthesis and Characterization of Ch NPs and Mechanistic Insights of Nanoparticle Formation

Ch NPs were synthesized via ionic gelation, a mild, aqueous, and solvent-free approach widely recognized for its simplicity and biocompatibility [10,11,13,14]. In contrast to conventional protocols that often require multistep processing or specialized equipment, the present study employed a simple dropwise addition system using a serological pipette–needle setup, enabling controlled introduction of TPP into the chitosan solution under continuous stirring. This approach produced a homogeneous milky suspension within minutes, indicating rapid nanoparticle formation and demonstrating a reproducible and accessible synthesis route.

Microscopic characterization supported these spectroscopic findings. SEM image showed aggregated nanoparticle clusters, whereas TEM micrographs revealed well-dispersed, nearly spherical particles with smooth surfaces. The spherical morphology observed here agrees with several earlier studies employing ionic gelation, where Ch NPs typically displayed uniform spherical shapes ranging from 50 to 150 nm [13,14]. This consistency supports the robustness of the electrostatic self-assembly process. The EDS spectra of the synthesized nanoparticles revealed predominant peaks of carbon, nitrogen, and oxygen, confirming the organic composition typical of chitosan-based materials. Similar elemental compositions were found in an earlier report [33].

Particle size analysis showed that nanoparticles with an average size of approximately 75 nm were observed by TEM, whereas DLS measurements revealed a larger hydrodynamic diameter (450 ± 8.7 nm) with a PDI of 0.45. This difference arises because DLS measures the hydrodynamic diameter of particles in suspension, which includes the hydrated polymeric layer surrounding nanoparticles, whereas TEM reflects the dry core size [12]. The PDI value indicates a moderately polydisperse system; although values below 0.5 are generally considered acceptable, some degree of size variability remains inherent to ionic gelation processes. In addition, the polymeric nature of chitosan promotes the formation of a hydrated shell and extended polymer chains in aqueous suspension, which can increase the apparent particle size measured by DLS. The relatively moderate polydispersity index (PDI ≈ 0.45) also suggests a degree of size distribution and potential interparticle interactions or aggregation, which may further contribute to the larger hydrodynamic diameter observed by DLS.

In contrast, the high positive zeta potential (+47.4 mV) indicates a strongly protonated nanoparticle surface and suggests excellent colloidal stability due to electrostatic repulsion between particles. Zeta potential values exceeding ±30 mV are widely regarded as indicative of stable nanoparticle dispersions [34]. Moreover, positively charged Ch NPs have been reported to interact strongly with negatively charged biological membranes, which may facilitate cellular association and uptake through electrostatic attraction [35].

Spectroscopic analyses confirmed the ionic interaction between chitosan and TPP. The UV–Vis absorption band at 321 nm corresponded to the typical surface plasmon resonance of Ch NPs, which generally occurs between 310 and 350 nm [34]. FTIR spectra showed characteristic peaks of chitosan at 3384 cm^−1^ (O–H/N–H stretching), 1639 cm^−1^ (amide I), and 1543 cm^−1^ (amide II), alongside new bands at 894 cm^−1^ (P–O–P asymmetric stretching) and 1094 cm^−1^ (PO_3_ stretching) from TPP. The appearance of a distinct band near 1541 cm^−1^ in Ch NPs confirmed the formation of N–H···O–P bonds, indicating electrostatic crosslinking between chitosan and TPP [5,36].

The XRD pattern of Ch NPs exhibited a broad halo centered at 2θ ≈ 20°, indicating a predominantly amorphous structure, consistent with previous reports [37]. Chitosan and its nanoparticle forms can exhibit crystalline, amorphous, or semi-crystalline structures depending on their molecular organization. In this study, the loss of crystallinity is attributed to ionic crosslinking between chitosan and TPP, which disrupts the native polymer packing [38]. The resulting amorphous structure is associated with increased molecular mobility and solubility, which may enhance interactions with biological systems [38,39].

**Figure 12:**
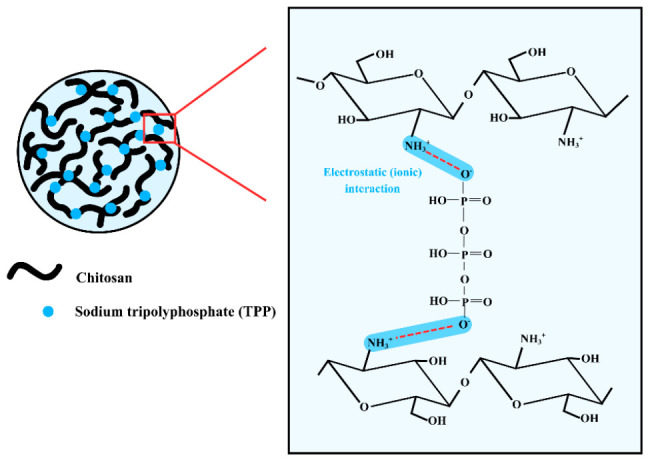
Mechanistic illustration of chitosan nanoparticle (Ch NP) formation via ionic gelation. Protonated amino groups (–NH_3_^+^) of chitosan interact electrostatically with negatively charged phosphate groups of TPP, forming ionotropic crosslinking networks. In addition to electrostatic interactions, hydrogen bonding interactions further stabilize the polymeric network. The combined interactions induce localized nucleation and subsequent self-assembly of chitosan chains into stable, nanoscale particles under mild aqueous conditions. The red dashed lines represent electrostatic interactions between protonated amino groups (–NH_3_^+^) of chitosan and negatively charged phosphate groups of TPP.

Mechanistically, nanoparticle formation is governed by ionic crosslinking between protonated amino groups (–NH_3_^+^) of chitosan and negatively charged phosphate groups of TPP [11,40,41]. Molecular-level studies further indicate that these interactions primarily occur through hydrogen-linked (H-link) and transverse (T-link) configurations, depending on the spatial orientation between interacting groups [11,42]. In the H-link configuration, electrostatic interactions occur within the same plane, whereas T-links involve interactions across different planes, contributing to structural heterogeneity within the network. Earlier computational work has also suggested additional configurations, such as M-link structures, although these are less consistently observed and may depend on specific modeling conditions [43]. As illustrated in Figure 12, TPP acts as a multivalent crosslinker bridging adjacent chitosan chains, forming a three-dimensional network stabilized by electrostatic interactions and hydrogen bonding. Collectively, these interaction modes give rise to a dynamic and heterogeneous crosslinked network that stabilizes nanoparticles during ionic gelation.

The nanoparticle formation process can be described as a hierarchical assembly in which initial ionotropic crosslinking promotes chain association, followed by the formation of nanoscale domains that aggregate into compact nanoparticle structures [43]. The dropwise addition of TPP enables gradual diffusion of the crosslinker, resulting in more controlled nucleation and reduced particle aggregation, thereby improving control over particle size distribution [12]. However, achieving highly uniform and stable nanoparticles via ionic gelation remains challenging due to the intrinsic mucoadhesive nature of chitosan and the sensitivity of the system to parameters such as pH, concentration, and mixing conditions [10,41]. Variations in precursor properties may further affect reproducibility and particle stability [11,14], and additional processing steps such as homogenization or filtration are sometimes required to improve uniformity [12]. In this context, the controlled dropwise addition strategy employed in the present study likely contributes to improved nucleation control and more consistent particle formation without the need for additional processing steps. Consistent with previous reports, an optimal chitosan-to-TPP ratio yields stable nanoparticles, whereas excess TPP may induce aggregation due to over-crosslinking [10,14].

Overall, the physicochemical properties obtained in this study are consistent with those reported for Ch NPs synthesized via ionic gelation, which typically exhibit nanoscale dimensions, positive surface charge, and moderate polydispersity. Importantly, the present approach combines simplicity, reproducibility, and controlled nanoparticle formation, making it a practical and scalable strategy for producing chitosan-based nanomaterials.

### 3.2. Antibacterial Performance of Synthesized Ch NPs Assessed by a Standardized Resazurin-Based Protocol

The antibacterial performance of Ch NPs was evaluated using a standardized resazurin-based microdilution assay, a sensitive and reproducible method for determining bacterial viability based on metabolic activity [44,45]. Under these conditions, Ch NPs exhibited a minimum inhibitory concentration (MIC) of 128 µg/mL against *Xoo*, whereas Bulk Ch showed no inhibition up to 512 µg/mL. The use of resazurin minimized interference from nanoparticle turbidity, enabling reliable assessment of antibacterial activity [45].

The antibacterial mechanism of Ch NPs is primarily attributed to electrostatic interactions between protonated amino groups (–NH_3_^+^) and negatively charged components of the bacterial outer membrane. These interactions can disrupt membrane integrity, increase permeability, and induce leakage of intracellular contents [1,4,19]. Additional mechanisms, such as chelation of essential cations and oxidative stress associated with reactive oxygen species (ROS), have also been reported, although they were not directly evaluated in this study [4,18]. The observed activity against *Xoo* is consistent with the known susceptibility of Gram-negative bacteria, whose outer membranes are rich in negatively charged lipopolysaccharides that facilitate strong electrostatic interactions with cationic nanoparticles [1,6].

Recent studies further indicate that antimicrobial stress can actively promote gene exchange processes [46]. Sublethal exposure to antibacterial agents has been shown to increase ROS levels, activate SOS response pathways, and alter membrane permeability, all of which contribute to enhanced plasmid-mediated conjugation and antibiotic resistance gene transfer [46]. These findings suggest that certain antibacterial treatments may unintentionally accelerate the spread of resistance genes within microbial communities.

In contrast, the antibacterial activity of Ch NPs is primarily mediated through physicochemical interactions at the cell surface rather than specific intracellular biochemical targets. This multi-target mode of action, involving electrostatic membrane disruption and increased permeability, has been widely reported for chitosan-based nanomaterials [4,17,19]. Unlike conventional antibiotics, which typically act on defined molecular targets and are therefore susceptible to resistance development, such surface-mediated mechanisms may reduce dependence on specific biochemical pathways associated with antimicrobial resistance [47]. In addition, nanomaterials possess unique physicochemical properties, including high surface area and multivalent interactions, which enable broad-spectrum antibacterial activity and may allow them to bypass conventional resistance mechanisms [48,49]. Consequently, Ch NPs may impose lower selective pressure for resistance development and horizontal gene dissemination, despite requiring higher concentrations than conventional antibiotics due to their distinct, surface-mediated mode of action [46].

Although nanoparticle-based treatments typically require higher concentrations than conventional antibiotics, their distinct mode of action and reduced reliance on target-specific interactions may limit the likelihood of resistance development and horizontal gene dissemination [48,49]. Therefore, Ch NPs represent a promising alternative strategy for managing bacterial plant pathogens such as *Xoo*, particularly in the context of sustainable agriculture where minimizing antimicrobial resistance is an increasing priority.

### 3.3. Phytotoxicity and Seed Germination Assay

The phytotoxicity of the synthesized Ch NPs was evaluated using a rice seed germination assay. The results indicate that Ch NPs did not adversely affect seed viability and were well tolerated under the tested conditions, as evidenced by the absence of visible phytotoxic symptoms and the consistently high GP across treatments (Table 2).

Beyond the absence of toxicity, Ch NP treatments were associated with improved germination dynamics and early seedling performance. The observed reductions in MGT and T_50_, together with increased GI and SVI, indicate a trend toward faster and more synchronized germination, particularly at 64 µg/mL. These effects suggest that Ch NPs may facilitate early germination processes rather than merely maintaining baseline germination performance, which is consistent with enhanced germination patterns and seedling development observed in other plant systems treated with Ch NPs [27,50].

The non-phytotoxic behavior observed in this study is consistent with previous reports indicating that chitosan-based nanomaterials are generally biocompatible and well tolerated in plant systems at appropriate concentrations [7,8,25,27,51]. However, their effects are concentration-dependent, as higher levels of nano-chitosan have been shown to inhibit plant growth and development, highlighting the importance of dosage optimization [27].

The mechanisms underlying the promotive effects of Ch NPs on seed germination are not fully understood, but several contributing factors have been proposed. At the physical level, Ch NPs may form a thin semi-permeable layer on the seed surface, which helps maintain moisture conditions favorable for germination. In addition, their nanoscale size enhances interaction with seed surfaces, resulting in greater adsorption compared to Bulk Ch, which may improve seed–nanoparticle contact [27]. More broadly, chitosan has been reported to interact with the seed coat and influence early physiological processes associated with germination [52].

At the physiological level, Ch NPs have been associated with stimulation of metabolic activity during germination, leading to enhanced seedling growth and vigor [50,53]. This includes improved antioxidant responses and modulation of oxidative stress, which are critical processes during early seedling establishment [53]. At the molecular level, Ch NPs have been reported to regulate plant growth through nutrient uptake and hormone-related pathways. For example, enhanced uptake of essential nutrients, including nitrogen, phosphorus, potassium, calcium, and magnesium, has been observed following treatment with Ch NPs [27]. In addition, activation of auxin-related pathways, particularly those associated with indole-3-acetic acid (IAA), has been suggested to contribute to improved seedling development [27,54].

Taken together, these findings indicate that the promotive effects of Ch NPs on germination likely result from a combination of surface interactions, metabolic activation, and physiological regulation rather than a single dominant mechanism. Importantly, the concentrations used in this study were selected based on antibacterial activity against *Xoo*, ensuring that plant exposure remained within effective antimicrobial levels. The results therefore demonstrate that Ch NPs can suppress bacterial growth while maintaining normal seed germination and early seedling development.

### 3.4. Biocompatibility Evaluation in Human Cell Lines

The biocompatibility of the synthesized Ch NPs was evaluated using HDF and HaCaT cell lines to assess their suitability for biological applications. Ch NPs maintained high cell viability (>90%) in both HDF and HaCaT cells at concentrations up to 128 µg mL^−1^, with only a slight, statistically insignificant reduction observed at the highest concentration (256 µg mL^−1^). These results indicate that the synthesized nanoparticles are non-cytotoxic and exhibit good biocompatibility in human skin-related cell models under the tested conditions. The observed increase in HDF cell viability at certain concentrations may be attributed to enhanced metabolic activity and/or an increase in the total number of viable cells, as the MTT assay reflects mitochondrial activity rather than direct cell counting. Therefore, this effect should be interpreted as an indication of non-cytotoxicity and potential support of normal cellular function, rather than a direct indication of a proliferative effect.

This observation is consistent with previous studies demonstrating that Ch NPs generally exhibit low cytotoxicity across diverse cell types. High cell viability has been reported in both human and mouse dermal fibroblasts [5,13], as well as human keratinocytes [55], following exposure to chitosan nanoparticles, supporting their overall biocompatibility. In addition, a comprehensive review has confirmed that Ch NPs exhibit low toxicity across a wide range of experimental systems, although their effects depend on particle characteristics and experimental conditions [56].

The favorable biocompatibility of Ch NPs can be attributed to both the intrinsic properties of chitosan and the physicochemical characteristics of the nanoparticles. Chitosan is a biodegradable and biologically compatible polymer that has been widely reported to exhibit low cytotoxicity across various cell types [56]. In addition, its nanoscale formulation provides a high surface area-to-volume ratio, enabling more efficient interaction with biological systems [5]. It is important to note that nanoparticle behavior in biological media may differ from that in deionized water due to interactions with serum proteins, leading to the formation of a protein corona. This can influence the effective hydrodynamic size and surface properties of nanoparticles in cell culture systems. In the present study, physicochemical characterization was performed in deionized water to provide a standardized baseline for comparison, while the cell-based assays were designed to evaluate biological responses under relevant exposure conditions.

Due to their positive surface charge, Ch NPs can interact electrostatically with negatively charged cell membranes, thereby facilitating cellular association and uptake [5]. Importantly, such interactions do not inherently induce cytotoxic effects at appropriate concentrations, as evidenced by studies demonstrating efficient cellular internalization of Ch NPs in fibroblast models without compromising cell viability [5]. These findings are consistent with the high cell viability observed in the present study and in other cell systems [13,55], indicating that nanoparticle–cell interactions can occur without triggering adverse cytotoxic responses.

Notably, previous studies have also highlighted that Ch NPs may exhibit selective biological effects, showing cytotoxic activity toward cancer cells while maintaining low toxicity in normal cells [57]. This selective behavior, together with their generally low cytotoxicity profile, has been reported across in vitro and in vivo studies [56]. However, the extent of toxicity may vary depending on factors such as concentration, exposure duration, particle size, and experimental conditions [57,58,59]. Therefore, careful consideration of these parameters is essential when evaluating the safety and application of Ch NPs.

### 3.5. Implications and Advantages of the Simplified Protocol

The simplified ionic gelation protocol developed in this study provides a practical and accessible approach for producing Ch NPs under mild aqueous conditions. The method is inexpensive and relies on readily available materials and a simple experimental setup, eliminating the need for specialized equipment. These features facilitate its implementation in laboratories with limited infrastructure.

Importantly, the combined results from antibacterial, phytotoxicity, and cell-based biocompatibility assays demonstrate that the synthesized Ch NPs exhibit a balance between biological activity and compatibility under the tested conditions. In particular, the observed enhancement of seed germination and early seedling performance suggests that Ch NPs may function as phytostimulatory or plant growth-promoting agents. At the same time, their ability to inhibit *Xoo* highlights their potential as nanobiocontrol agents for managing phytopathogenic bacteria in agricultural systems.

In addition, the high cell viability observed in human-derived cell lines, together with the indication of proliferative responses in HDF cells at certain concentrations, suggests that Ch NPs can interact with mammalian cells without inducing significant cytotoxic effects under the tested conditions. These findings support their potential use in cell-based applications, such as delivery systems or wound-related applications, where cellular compatibility is required. However, further in vivo and long-term studies are necessary to validate these possibilities in more complex biological systems.

## 4. Materials and Methods

### 4.1. Chemicals and Reagents

Low-molecular-weight chitosan (50–190 kDa, degree of deacetylation 75–85%, catalog no. 448869) was purchased from Sigma-Aldrich (St. Louis, MO, USA). TPP (≥85%, catalog no. 238503) was obtained from Merck (Sigma-Aldrich, St. Louis, MO, USA). Glacial acetic acid (≥99.7%) and sodium hydroxide (NaOH, analytical grade) were obtained from QRëC (Auckland, New Zealand). Absolute ethanol (≥99.9%) was purchased from QRëC (Auckland, New Zealand).

Resazurin sodium salt (Sigma-Aldrich, USA) was used as a viability indicator in antibacterial assays. Streptomycin sulfate (Sigma-Aldrich, USA) was used as a positive control.

All aqueous solutions were prepared using deionized water (resistivity 18.2 MΩ·cm) produced by a Milli-Q purification system (Millipore, Burlington, MA, USA).

### 4.2. Simple Dropwise Ionic-Gelation Synthesis of Ch NPs

Ch NPs were synthesized using a modified dropwise ionic-gelation method adapted from Rasool et al. [60], with procedural modifications to enable controlled manual dropwise addition using a gravity-assisted serological pipette system.

All glassware used for nanoparticle synthesis was soaked overnight in 10% (*v*/*v*) nitric acid, rinsed thoroughly with deionized (DI) water, and dried at 60 °C prior to use to minimize inorganic contamination that could interfere with electrostatic crosslinking.

#### 4.2.1. Preparation of Chitosan Solution

Chitosan solution (0.1% *w*/*v*) was prepared by accurately weighing 0.1 g of low-molecular-weight chitosan (50–190 kDa, 75–85% degree of deacetylation) using an analytical balance (Mettler Toledo, XS204,Greifensee, Switzerland), and it was slowly added to 100 mL of 1% (*v*/*v*) acetic acid in a 250 mL borosilicate beaker.

The suspension was stirred at 500 rpm using a magnetic stirrer (Daihan Labtech Co., Ltd., LMS-1003, Namyangju, Republic of Korea) at room temperature (25 ± 2 °C) for 24 h until a clear, slightly viscous solution was obtained. Complete dissolution was confirmed visually by the absence of visible particulates and gel aggregates. The solution was filtered through a 0.22 µm polyethersulfone (PES) syringe filter (Sartorius, Göttingen, Germany) to remove undissolved residues. Filtration was performed slowly to prevent shear-induced bubble formation. The pH was adjusted dropwise using 1 M NaOH under continuous stirring until reaching pH 5.5 ± 0.1, monitored using a calibrated digital pH meter (Fisher Scientific, Accumet^®^ Basic AB15, Waltham, MA, USA). The solution was allowed to equilibrate for 30 min after pH adjustment before proceeding to nanoparticle formation.

#### 4.2.2. Preparation of TPP Crosslinker Solution

TPP (0.25 g) was dissolved in 100 mL DI water to prepare a 0.25% (*w*/*v*) solution. The solution was stirred for 30 min until fully dissolved and subsequently filtered through a 0.22 µm polyethersulfone (PES) syringe filter (Millipore, USA). Both chitosan and TPP solutions were freshly prepared prior to each synthesis batch to ensure reproducibility.

#### 4.2.3. Assembly of the Manual Dropwise Addition System

To achieve controlled addition without peristaltic pumps or homogenizers, a gravity-assisted dropwise delivery system was constructed.

A sterile 10 mL serological pipette (SPL Life Sciences, Pocheon, Korea) was modified by trimming the upper end to facilitate filling with TPP solution. The lower dispensing end was securely fitted with a 24G hypodermic needle (internal diameter 0.55 mm; Nipro Corporation, Osaka, Japan). The joint was sealed using Parafilm to prevent leakage. The pipette–needle assembly was mounted vertically on a retort stand (Thermo Fisher Scientific, USA) and positioned such that the needle tip was 2 cm above the surface of the chitosan solution Figure 1A,B.

The chitosan solution (100 mL) was placed in a 250 mL beaker on a magnetic stirrer and stirred at a constant speed of 500 rpm to create a stable vortex approximately 1 cm in depth. The dropwise addition rate was maintained at approximately 1 drop per second (~1 mL/min). The full 100 mL of TPP solution was added over 10–15 min.

Critical parameter: The needle tip must remain centered within the vortex region to ensure immediate dispersion of each droplet and prevent localized over-crosslinking, which can result in aggregation.

#### 4.2.4. Nanoparticle Formation and Post-Addition Stabilization

During dropwise addition, the solution gradually transitioned from transparent to opalescent and finally to a uniform milky suspension, indicating nanoparticle formation.

After complete addition of TPP, stirring was continued for 2 h at room temperature to allow complete ionotropic crosslinking between protonated –NH_3_^+^ groups of chitosan and negatively charged phosphate groups of TPP. The final chitosan-to-TPP mass ratio was 1:1. The suspension was allowed to stand for 30 min prior to purification to stabilize electrostatic interactions.

#### 4.2.5. Purification and Washing

The nanoparticle suspension was transferred into 50 mL centrifuge tubes and centrifuged at 5000 rpm (≈3000× *g*) for 10 min at 4 °C using a refrigerated centrifuge (NÜVE, model NF 800, Ankara, Turkey). The supernatant was carefully removed without disturbing the pellet. The pellet was resuspended in 40 mL of DI water by gentle vortexing and centrifuged again under identical conditions. This washing step was repeated three times to remove unreacted TPP and acetic acid. Subsequently, the pellet was washed twice with absolute ethanol to facilitate dehydration and reduce capillary forces during drying.

#### 4.2.6. Freeze-Drying and Powder Preparation

The washed nanoparticle suspension was frozen at −80 °C for 12 h and then lyophilized using a freeze dryer (Martin Christ Gefriertrocknungsanlagen GmbH, Gamma 2-16 LSC, Osterode am Harz, Germany) at −50 °C and 0.04 mbar for 48 h. The dried cake was gently ground using an agate mortar and pestle to obtain a fine powder. The final yield was recorded, and the powder was stored in amber glass vials in a desiccator at room temperature.

### 4.3. Physicochemical Characterization of Ch NPs

The synthesized Ch NPs were characterized in terms of morphology, particle size distribution, hydrodynamic diameter, surface charge, optical properties, and functional group interactions prior to biological evaluation.

For ESEM (Thermo Fisher Scientific, Quattro S ESEM, Hillsboro, OR, USA), freeze-dried nanoparticle powder was dispersed in sterile deionized water using ultrasonic agitation for 10–15 min to obtain a homogeneous suspension. A drop of the suspension was placed onto carbon tape mounted on aluminum stubs and air-dried at room temperature. The samples were sputter-coated with a thin layer of gold prior to imaging to improve conductivity. Surface morphology and elemental analysis were examined using a field-emission scanning electron microscope (FE-SEM) (JEOL Ltd., JSM-7800F, Tokyo, Japan) equipped with an EDS detector. Images were acquired at an accelerating voltage of 5–10 kV under high-vacuum conditions.

Transmission electron microscopy (TEM) was performed to determine particle morphology and primary particle size. A diluted aqueous suspension of Ch NPs (approximately 0.05 mg/mL) was prepared by sonication to minimize aggregation. A 10 µL aliquot was deposited onto a carbon-coated copper grid and allowed to dry at room temperature. Micrographs were obtained using a transmission electron microscope (Thermo Fisher Scientific, Talos F200X G2, Hillsboro, OR, USA) operating at 200 kV. Particle dimensions were measured from representative images (n > 100 particles) using ImageJ software (version 1.53t, National Institutes of Health, Bethesda, MD, USA) to calculate the average particle size and construct size distribution profiles.

The hydrodynamic diameter and PDI of Ch NPs in aqueous suspension were determined by dynamic light scattering (DLS) using a Zetasizer Nano ZS instrument (Malvern Instruments Ltd., Nano S90, Malvern, UK). Nanoparticle suspensions were appropriately diluted in deionized water to avoid multiple scattering and equilibrated at 25 °C prior to measurement. Each sample was measured in triplicate, and the results were expressed as mean ± standard deviation. Zeta potential measurements were performed using the same instrument to assess surface charge and colloidal stability at 25 °C in folded capillary cells under automatic voltage settings.

The optical properties of Ch NPs were analyzed by UV–visible spectroscopy. The nanoparticles were dispersed in deionized water using an ultrasonic homogenizer to ensure uniform suspension and then serially diluted as required. Aliquots were transferred into quartz cuvettes for spectral analysis. UV–visible absorption spectra were recorded over the wavelength range of 200–800 nm using a UV–Vis spectrophotometer (Analytik Jena GmbH + Co. KG, SPECORD 200 PLUS, Jena, Germany). Deionized water was used as a blank control.

Fourier transform infrared (FTIR) spectroscopy was conducted to confirm functional groups and ionic crosslinking interactions between chitosan and TPP. Spectra of Bulk Ch and freeze-dried Ch NPs were recorded using an FTIR spectrometer (Bruker, Tensor 27, Ettlingen, Germany) equipped with an attenuated total reflectance (ATR) accessory. Measurements were performed over a spectral range of 4000–400 cm^−1^ at a resolution of 4 cm^−1^ with 32 scans per sample. Characteristic absorption bands corresponding to amide I, amide II, and phosphate-related vibrations were analyzed to confirm electrostatic interactions responsible for nanoparticle formation.

### 4.4. Determination of Antibacterial Activity Against Xoo by Broth Microdilution Assay

The antibacterial activity of Ch NPs against *Xoo* was evaluated using a broth microdilution assay to determine the minimal inhibitory concentration (MIC), following the Clinical and Laboratory Standards Institute (CLSI) [61] guidelines with minor modifications. Resazurin was used as a metabolic viability indicator as previously described by Sarker et al. [44]

The *Xoo* isolate used in this study was previously obtained from naturally infected rice seeds of the susceptible cultivar KDML105 and characterized as described in our earlier report [62]. Briefly, the bacterium was isolated on peptone sucrose agar (PSA) medium and exhibited typical yellow, mucoid colony morphology and biochemical characteristics consistent with *Xoo*. Pathogenicity was confirmed using the leaf-clipping inoculation method on rice plants [62]. For antibacterial assays, the isolate was maintained on PSA at 28 °C and subcultured in peptone sucrose broth (PSB) prior to use.

For inoculum preparation, a single well-isolated colony from a 48–72 h PSA plate was transferred into 10 mL PSB and incubated at 28 °C with shaking at 150 rpm for 24 h to obtain cells in the logarithmic growth phase. The bacterial suspension was adjusted to approximately 1 × 10^6^ CFU/mL based on optical density calibration and confirmed by serial dilution plating when required.

Ch NPs were prepared as a sterile suspension at an initial concentration of 512 µg/mL in sterile deionized water and dispersed by sonication under cold conditions (approximately 4 °C) for 15 min to ensure homogeneous dispersion. Streptomycin sulfate was used as a reference antibiotic control and prepared at an initial concentration of 64 µg/mL.

For MIC determination, 100 µL of PSB was dispensed into each well of a sterile 96-well microplate, except for column 2. Subsequently, 200 µL of the nanoparticle suspension was added to column 2, and a two-fold serial dilution was performed from column 2 to column 11 using a multichannel micropipette to generate a concentration gradient across the plate. After dilution, 10 µL of the standardized bacterial inoculum was added to each well except columns 1 and 2. Column 1 received 10 µL of sterile deionized water and served as the negative control, while column 12 was designated as the positive growth control (Figure 6).

The microplate was incubated statically at 28 °C for 48 h. Following incubation, 20 µL of resazurin solution (0.2 mg/mL) was added to each well except column 2, and the plate was further incubated at 28 °C for 4 h. Bacterial viability was evaluated based on the colorimetric change in resazurin. The MIC endpoint was determined visually without the use of instrumental measurements, based on the observed color transition in the wells. A transition from blue to pink indicated active bacterial growth, whereas retention of blue or purple coloration indicated inhibition of bacterial growth [45]. The MIC was defined as the lowest concentration of Ch NPs that prevented color change and indicated complete inhibition of visible bacterial growth. All experiments were performed in three independent biological replicates.

### 4.5. Phytotoxicity Assessment by Rice Seed Germination Assay

The phytotoxicity of Ch NPs was evaluated using a rice seed germination assay adapted from Mahakham et al. [63] with modification by using soft agarose instead of filter paper to minimize moisture condensation on the lid of the Petri dish. Rice seeds (*Oryza* ‘KDML105’) were kindly provided by the Khon Kaen Rice Research Center. Prior to experimentation, seeds were dried at 45 °C for 3–4 days to reduce moisture content to approximately 12–14%, ensuring a uniform physiological state.

Surface sterilization was performed using 1% (*v*/*v*) sodium hypochlorite (NaClO) for 10 min with gentle agitation to remove surface contaminants. After sterilization, the seeds were rinsed three times with sterile distilled water and once with sterile deionized (DI) water to eliminate residual disinfectant. Equal quantities of seeds were then transferred into sterile 50 mL centrifuge tubes for subsequent treatment.

Ch NPs were prepared as sterile suspensions at concentrations corresponding to 0.5× MIC (64 µg/mL) and 1× MIC (128 µg/mL), as determined in the antibacterial assay. The suspensions were dispersed using an ultrasonic bath under cold conditions (approximately 4 °C) for 15 min to ensure homogeneous distribution. Rice seeds were incubated in the nanoparticle suspensions at a seed-to-solution ratio of 1:4 (g/mL). Seeds soaked in sterile DI water without nanoparticles served as the control. Incubation was performed in the dark at 25 °C under continuous aeration using a rocking shaker (MIULAB Co., Ltd., models HS-25, Hangzhou, China) at 50 rpm for 24 h.

Following incubation, the seeds were rinsed three times with sterile DI water and gently blotted dry using sterile tissue paper. The treated seeds were then placed on 90 × 15 mm sterile Petri dishes containing 0.4% (*w*/*v*) agarose gel as the growth medium. Ten seeds were arranged per dish at equal spacing to prevent physical interference during germination. The dishes were sealed with Parafilm M to maintain humidity and incubated in a temperature-controlled chamber at 25 ± 2 °C.

Seed germination was monitored daily for 7 consecutive days. A seed was considered germinated when the radicle length exceeded 5 mm. Germination parameters, including GP, GE_3_, MGT, T_50_, GI, GVe, GV, SVI, and SL/RL, were calculated based on established equations reported by Coolbear et al. [64], Feizi et al. [65], and Allam et al. [53]; the equations are provided in Table S1 (Supplementary Materials).

At the end of the experimental period, root and shoot lengths of rice seedlings were measured using a digital caliper, and representative images were recorded using a digital camera. All treatments were performed in triplicate.

### 4.6. Biocompatibility Assessment by MTT Assay

The biocompatibility of Ch NPs was evaluated using primary HDF (ATCC^®^ PCS-201-012™ [66], American Type Culture Collection, Manassas, VA, USA) and HaCaT cells (Cat. No. T0020001, AddexBio, San Diego, CA, USA). Cells were cultured in Dulbecco’s Modified Eagle Medium (DMEM; Cat# 11965-092, Gibco, Thermo Fisher Scientific, Waltham, MA, USA) supplemented with 10% fetal bovine serum (FBS; Cat# 10270-106, Gibco) and 1% penicillin–streptomycin (100 U/mL penicillin and 100 µg/mL streptomycin; Cat# 15140-122, Gibco). Cells were maintained under standard culture conditions at 37 °C in a humidified incubator with 5% CO_2_ atmosphere.

Upon reaching approximately 80% confluence, cells were detached using TrypLE™ Express Enzyme (1X, no phenol red; Cat. No. 12604021, Gibco, Thermo Fisher Scientific, Waltham, MA, USA) and counted using a hemocytometer. Cells were seeded into sterile 96-well plates at a density of 1 × 10^4^ cells per well and allowed to attach for 24 h prior to treatment.

Ch NPs were prepared as sterile suspensions in complete culture medium at various concentrations corresponding to the antibacterial test range and were sonicated briefly to ensure uniform dispersion. After 24 h of cell attachment, the culture medium was replaced with fresh medium containing Ch NPs at different concentrations, and the cells were incubated for 24 h. Untreated cells cultured in complete medium served as the negative control.

Cell viability was assessed using a modified MTT colorimetric assay based on a previously described method [67]. Briefly, MTT reagent (5 mg/mL in PBS) was diluted in DMEM at a ratio of 1:10, and 100 µL of this mixture was added to each well. The plates were incubated at 37 °C for 4 h to allow mitochondrial dehydrogenases in viable cells to reduce MTT to insoluble formazan crystals. After incubation, the medium was carefully aspirated, and the formed formazan crystals were dissolved by adding 100 µL of dimethyl sulfoxide (DMSO) to each well. Absorbance was measured at 570 nm with background correction at 630 nm using a microplate reader (Molecular Devices, SpectraMax M5, San Jose, USA). The corrected absorbance value was calculated by subtracting the 630 nm reading from the 570 nm reading.

Cell viability was expressed as a percentage relative to untreated control cells using the following equation:


C=[(Absorbance of treated cell)/(Absorbance of control cell)]×100×

All experiments were performed in three independent biological replicates, and results were expressed as mean ± standard deviation (SD).

### 4.7. Statistical Analysis

All experimental data were expressed as mean ± standard deviation. Statistical analyses were performed using IBM SPSS Software (version 28, IBM Corp., Armonk, NY, USA). Differences among treatment groups were evaluated using one-way analysis of variance (ANOVA) followed by Duncan’s multiple range test. Statistical significance was defined at *p* < 0.05.

## 5. Conclusions

This study demonstrates a simplified and inexpensive ionic gelation method for the reproducible synthesis of Ch NPs using a dropwise approach under mild aqueous conditions. The method relies on readily available materials and a simple experimental setup, enabling consistent nanoparticle formation without the need for specialized equipment. The synthesized Ch NPs exhibited effective antibacterial activity against *Xoo*, while maintaining normal seed germination and early seedling development, together with high cell viability in human-derived cell lines under the tested conditions. These results indicate that Ch NPs can achieve a balance between biological activity and compatibility across microbial, plant, and cell-based systems. In addition, this work establishes a multi-system evaluation approach by integrating antibacterial, phytotoxicity, and cell-based biocompatibility assays under aligned experimental conditions, enabling a more comprehensive assessment of nanoparticle performance. Overall, the combination of a simplified synthesis method and an integrated bioassay framework provides a practical and resource-efficient basis for evaluating chitosan-based nanomaterials and supports their further development for applications in agriculture and related biological systems.

## Figures and Tables

**Table 1:** Particle size, Polydispersity Index (PDI), and zeta potential value of the synthesized Ch NPs.

Sample No.	Particle Size (nm)	PDI	Zeta Potential (mV)
1	460.9	0.426	47.5
2	446.0	0.448	48.3
3	445.5	0.468	46.3
Mean ± SD	450.80 ± 8.75	0.45 ± 0.02	47.37 ± 1.01

**Table 2:** Effects of Bulk Ch and Ch NPs on germination performance, germination speed, and seedling vigor of rice seeds. Values are presented as mean ± standard deviation (SD) of three replicates (*n* = 3). Different superscript letters within the same column indicate significant differences among treatments according to one-way analysis of variance (ANOVA) followed by Duncan’s multiple range test at *p* < 0.05. GP was 100% in all treatments and therefore showed no significant differences.

Treatment	Germination		Germination Speed					Seedling Vigor/Growth		
	GP	GE_3_ (%)	MGT (Days)	T_50_ (Days)	GI	GVe	GV	Seedling Length (cm)	SVI	SL/RL
Control	100	66.67 ± 5.77^b^	3.37 ± 0.15^b^	2.74 ± 0.09^b^	3.23 ± 0.40^b^	100.84 ± 9.50^b^	30.16 ± 2.75^b^	12.70 ± 2.07^b^	1270 ± 207^b^	0.75 ± 0.10^b^
BC 64 µg/mL	100	90.00 ± 10.00^a^	2.03 ± 0.57^a^	1.57 ± 0.74^a^	6.62 ± 1.52^a^	183.45 ± 36.24^a^	66.67 ± 29.74^a^	14.85 ± 0.52^b^	1485 ± 52^b^	0.96 ± 0.05^a^
BC 128 µg/mL	100	90.00 ± 10.00^a^	1.70 ± 0.26^a^	0.79 ± 0.07^a^	7.78 ± 0.51^a^	209.45 ± 13.58^a^	90.48 ± 8.25^a^	19.07 ± 0.87^a^	1907 ± 87^a^	0.72 ± 0.08^b^
Ch NPs 64 µg/mL	100	93.33 ± 5.77^a^	1.63 ± 0.29^a^	0.68 ± 0.05^a^	8.28 ± 0.48^a^	219.06 ± 13.18^a^	104.76 ± 8.25^a^	16.14 ± 1.62^a^	1614 ± 162^a^	0.89 ± 0.05^a^
Ch NPs 128 µg/mL	100	86.67 ± 5.77^a^	2.13 ± 0.15^a^	1.25 ± 0.25^a^	6.46 ± 0.29^a^	179.17 ± 7.30^a^	61.90 ± 8.25^b^	16.92 ± 0.33^a^	1692 ± 33^a^	0.77 ± 0.03^b^

## Data Availability

The data presented in this study are available in this article and the Supplementary Materials. Further inquiries can be directed to the corresponding authors.

## Supplementary Materials

The following supporting information can be downloaded at: https://www.mdpi.com/article/10.3390/ijms27083673/s1.
