# Supplementary material for: A Simple Ionic-Gelation Method for Chitosan Nanoparticle Synthesis and Standardized Protocols for Biological Safety Assessment: Antibacterial Activity, Phytotoxicity, and Biocompatibility
Source: Int J Mol Sci. 2026 Apr 20;27(8):3673. doi: 10.3390/ijms27083673 (PMC13116750; doi:10.3390/ijms27083673)
Supplement: Supplementary file 1 [file ijms-27-03673-s001.zip › ijms-4249880-supplementary.pdf]

International Journal of Molecular Sciences

Supplementary Materials

A Low-Cost and Simple Ionic-Gelation Method for Chitosan Nanoparticle Synthesis and Standardized Protocols for Biological Safety Assessment: Antibacterial Activity, Phytotoxicity, and Biocompatibility

Kanchit Rahaeng [1], Atcha Oraintara [2] and Wuttipong Mahakham [1,*]

[1] Department of Biology, Faculty of Science, Khon Kaen University, Khon Kaen 40002, Thailand; kanchit.ra@kkumail.com (K.R.); mwuthi@kku.ac.th (W.M.)
[2] Department of Microbiology, Faculty of Science, Khon Kaen University, Khon Kaen 40002, Thailand; atcha@kku.ac.th (A.O.)
* Correspondence: mwuthi@kku.ac.th (W.M.)

Table S1. Germination and seedling growth indices with corresponding formulas and symbol definitions.

No.	Parameters (Abbrev.) and Unit	Formula	Description (symbol definitions included)	References
1	Germination Percentage (GP)<br><br>Unit: %	$GP = \frac{Number\ of\ germinated\ seeds}{Total\ seeds} \times 100$	Indicates total germination success. Number of germinated seeds = cumulative count at the final day; Total seeds = number tested per replicate.	Allam et al. (2024)
2	Germination Energy (GE_3)<br><br>Unit: %	$GE_{d3} = \frac{Seeds\ germinated\ within\ 3\ days}{Total\ seeds} \times 100$	Early vigor index showing metabolic activation within the first 72 h. Seeds germinated within 3 days = cumulative count at day 3.	Adapted from Allam et al. (2024)

 https://doi.org/10.3390/ijms27083673

Table S1. (Cont.)

No.	Parameters (Abbrev.) and Unit	Formula	Description (symbol definitions included)	References
3	Mean Germination Time (MGT)<br><br>Unit: days	$MGT = \frac{\sum(F \times X)}{\sum F}$	Average time required for a seed to germinate. F = number of new germinated seeds on day X; X = day number.	Feizi et al. (2013)
4	Time to 50% Germination (T_{50})<br><br>Unit: days	$T_{50} = t_i + \left[\frac{(N/2) - n_i}{n_j - n_i}\right] \times (t_j - t_i)$	Time (days) required for 50% of total seeds to germinate. N = total seeds, n_i = number germinated before 50%, n_j = after 50%, t_i, t_j = corresponding times. Lower T_{50} indicates faster and more uniform germination.	Coolbear et al. (1984)
5	Germination Index (GI)<br><br>Unit: -	$GI = \sum \frac{E_t}{D_t}$	Combines rate and uniformity. E_t = newly emerged seeds on day t; D_t = corresponding day number.	Allam et al. (2024)
6	Germination Velocity (GVe)<br><br>%•day^{-1}	$GVe = \sum \frac{GP_t}{t}$	Average rate of germination per day. GP_t = cumulative germination (%) on day t; t = elapsed days.	Allam et al. (2024)
7	Germination Value (GV)<br><br>Unit: –	$GV = PV \times MDG$	Composite index summarizing speed and uniformity of germination.	Feizi et al. (2013)

https://doi.org/10.3390/ijms27083673

Table S1. (Cont.)

No.	Parameters (Abbrev.) and Unit	Formula	Description (symbol definitions included)	References
8	Seedling Shoot Length (SL)<br><br>Unit: cm	Measured directly	Mean shoot length per seedling (coleoptile region).	Allam et al. (2024)
9	Seedling Vigor Index I (SVI-I)<br><br>Unit: –	$SVI = L_{seedling} \times GP_{final}$	Integrates seedling growth and germination success. L(seedling) = total seedling length (shoot + root, cm); GP(final) = final germination (%).	Allam et al. (2024)
10	Shoot–Root Ratio (SRR)<br><br>Unit: –	$SRR = \frac{SL}{RL}$	Indicates resource allocation between shoot and root. SL = shoot length; RL = root length.	Allam et al. (2024)

Disclaimer/Publisher's Note: The statements, opinions and data contained in all publications are solely those of the individual author(s) and contributor(s) and not of MDPI and/or the editor(s). MDPI and/or the editor(s) disclaim responsibility for any injury to people or property resulting from any ideas, methods, instructions or products referred to in the content.

https://doi.org/10.3390/ijms27083673
